# Survival Outcomes Associated with First and Second-Line Palliative Systemic Therapies in Patients with Metastatic Bladder Cancer

**DOI:** 10.3390/curroncol28050325

**Published:** 2021-09-29

**Authors:** Arshia Beigi, Saba Vafaei-Nodeh, Longlong Huang, Shaun Z. Sun, Jenny J. Ko

**Affiliations:** 1 Department of Medicine, University of British Columbia, Vancouver, BC V6T 1Z3, Canada; abeigi@student.ubc.ca; 2 Faculty of Medicine, University of British Columbia, Vancouver, BC V6T 1Z3, Canada; sabavk@student.ubc.ca; 3 Faculty of Mathetmatics and Statistics, University of the Fraser Valley, Abbotsford, BC V2S 7MH, Canada; longlong.huang@ufv.ca (L.H.); shaun.sun@ufv.ca (S.Z.S.); 4 Department of Medical Oncology, British Columbia Cancer, Abbotsford, BC V2S 0C2, Canada

**Keywords:** metastatic bladder cancer, chemotherapy, immunotherapy, survival, real-world data

## Abstract

Background: Real-world data on palliative systemic therapies (PST) in treating metastatic bladder cancer (mBC) is limited. This study investigates current trends in treating mBC with first- (1L) and second-line (2L) chemotherapy (CT) and immunotherapy (IT). Methods: A chart review was conducted on patients diagnosed with stage II-IV bladder cancer in 2014–2016. Survival outcomes were compared between chemotherapy, immunotherapy, and supportive care. Results: out of 297 patients, 77% were male. 44% had stage IV disease at diagnosis. Median age at metastasis was 73 years. 40% of patients received 1L PST and 34% received 2L PST. Median overall survival (mOS) was longer in those receiving PST versus no treatment (*p* < 0.001). Patients receiving CT and IT sequentially had the longest mOS (18.99 months). First-line IT and CT mOS from treatment start dates were 5.03 and 9.13 months, respectively (*p* = 0.81). Gemcitabine with cisplatin (8.88 months) or carboplatin (9.13 months) were the most utilized 1L chemotherapy regimens (*p* = 0.85). 2L IT and CT mOS from treatment start dates were 6.72 and 3.78 months, respectively (*p* = 0.15). Conclusion: real-world mOS of >1.5 years in mBC is unprecedented and supports using multiple lines of PST. Furthermore, immunotherapy may be a comparable alternative to chemotherapy in both 1L and 2L settings.

## 1. Introduction

Bladder cancer is the most common malignancy of the urinary system, and the ninth most common in the world [1]. In 2020, an estimated 81,400 newly diagnosed cases of bladder cancer and 17,980 associated deaths were expected in the US [2]. Approximately 5% of new cases of bladder cancer are metastatic at diagnosis, and half of the patients diagnosed with muscle-invasive disease develop metastasis within two years [3,4]. The overall 5-year survival for any stage bladder cancer is 76.9%, however, metastatic disease carries a much worse prognosis with a 5-year survival rate of only 5.5% [4].

Approximately 90% of bladder cancers diagnosed in developed countries are classified as urothelial carcinoma otherwise known as transitional cell carcinoma [5]. The recommended first-line regimen for the treatment of metastatic urothelial bladder cancer involves cisplatin-containing combination chemotherapy, such as gemcitabine and cisplatin or methotraxate, vinblastine, doxorubicin, and cisplatin (both level 1b evidence) [6]. However, up to 50% of patients with metastatic bladder cancer are ineligible for cisplatin-based treatments [7]. This can be due to either poor performance status (ECOG ≥ 2), other comorbidities (including kidney disease, neuropathy, hearing difficulties, etc) or inability to tolerate the adverse effects of therapy [7]. Patients that are ineligible for cisplatin-based therapies may benefit from carboplatin in combination with gemcitabine instead (level 2a) [6].

With disease progression on chemotherapy, immunotherapy is often offered in the second-line setting. These FDA-approved immune-checkpoint inhibitors include anti-programmed cell death-1 (PD-1) antibodies (e.g., pembrolizumab and nivolumab) and anti-programmed cell death ligand-1 (PD-L1) antibodies (e.g., atezolizumab, durvalumab and avelumab). Currently, pembrolizumab is the only immunotherapy agent with level 1b evidence for use in second-line setting; however, alternative agents such as atezolizumab and nivolumab (level 2a) have also been used. Immunotherapy may be used in a first-line setting in either cisplatin-ineligible patients with high PD-L1 expression or those ineligible for any platinum-based therapies (level 2a) [6]. Recently, a phase III clinical trial has demonstrated a significant increase in overall survival using avelumab as maintenance therapy in individuals with metastasis who have completed platinum-based therapy and have achieved at least a stable disease [8].

Limited data exist to show the benefit of immunotherapy in real-world patients with locally advanced unresectable or metastatic bladder cancer who may otherwise not have been able to enroll in or qualify for clinical trials. Considering all of the recent advances in the treatment of metastatic bladder cancer (mBC), we sought to understand the use and effectiveness of various palliative systemic therapies (PSTs) in the real-world setting. Therefore, the purpose of this study is to report on patterns of treatment provided to patients with mBC as well as to compare survival outcomes of immunotherapy and chemotherapy using a population-based database.

## 2. Materials and Methods

### 2.1. Study Design and Patient Population

Approval was obtained from the Research Ethics Board to conduct a retrospective chart review of all patients in the BC Cancer database diagnosed with stage II-IV bladder cancer between 1 January 2014 to 31 December 2016. Data was collected from the date of bladder cancer diagnosis until 1 July 2020, date of death, or the last date of follow-up, whichever occurred first. Patients with metastatic disease were then identified and information was collected on patient demographics, tumor histopathology (grade, histology, presence of lymphovascular invasion), disease characteristics, as well as initial and subsequent treatments provided. Patients were staged using the American Joint Committee on Cancer (AJCC) 8th Edition TNM system [9]. Stage IV disease was defined as metastatic disease to distant lymph nodes (IVA) and/or visceral organs (IVB). Staging was based on pathology reports from transurethral resection of bladder tumor (TURBT) or radical cystectomy procedures, as well as imaging reports including computed tomography, magnetic resonance imaging, nuclear medicine, and positron emission tomography. Survival outcomes were compared between treatment modalities from the date of metastasis. To further understand the efficacy of treatments, outcomes were also compared in first-line (1L) and second-line (2L) settings. Patients were excluded if they received 1L or 2L agents as part of a clinical trial. Furthermore, patients were excluded from 1L analysis if they were diagnosed with metastasis in the last six months of data collection. Additionally, patients were excluded from 2L analysis if 1L therapy was terminated within the last six months of data collection.

### 2.2. Clinical Outcomes

In the overall analysis, patient survival outcomes were compared between those receiving immunotherapy (IT) only, chemotherapy (CT) only, both chemotherapy and immunotherapy and no treatment (NT). Overall survival (OS) was defined as the time from date of metastasis to the earliest of death or last follow-up.

In the 1L analysis, median OS (mOS) was compared between IT or CT versus NT with OS defined as the time from date of metastasis to the earliest of death or last follow-up. Subsequently, we compared clinical outcomes within the cohorts of patients undergoing (a) 1L therapy, including IT versus CT, and (b) 1L platinum-based combination chemotherapy, including gemcitabine with cisplatin (GCis) or carboplatin (GCarb). The primary outcome here was OS, defined as the time from the initiation of therapy to the earliest of death or last follow-up.

Similarly, in the 2L setting, OS analysis was conducted for patients receiving treatment (IT or CT) versus NT. Here, OS was defined as the time from termination date of 1L therapy to the earliest of death or last follow-up. Clinical outcomes were also directly compared in patients treated with 2L IT versus CT. The primary outcome was OS, defined as the time from the initiation of 2L therapy to the earliest of death or last follow-up.

### 2.3. Statistical Analysis

Statistical analysis was performed using the R software (version 3.6.3). All of the reported *p*-values were two-sided and a value of <0.05 was considered statistically significant. The primary analysis used log-rank test to compare overall survival between groups. Overall survival curves were constructed using the Kaplan–Meier method. Treatment effects were estimated from Cox regression analyses when proportional hazards could be assumed. If this assumption, however, was violated, then the restricted mean survival time was used to estimate survival differences over a follow-up period of three or four years based on the minimum survival time.

## 3. Results

A total of 297 patients with mBC were included in this study. Table 1 summarizes baseline patient, disease, and treatment characteristics. The majority of the patient population was male (77%). The median age at metastasis was 73 years. Most patients had a high-grade tumor (92%). Histologies included urothelial (78%), mixed (15%), neuroendocrine (4%), squamous (2%) and glandular (<1%). Cancer stage at initial presentation consisted of: II (25%), III (31%), and IV (44%). In this patient cohort, 46% received radical cystectomy and 25% received curative chemotherapy prior to metastasis. 

Of note, each of the characteristics in Table 1 were compared between the three treatment groups (IT only, CT only and both CT and IT). There were no significant differences in terms of basic patient demographics or tumor histopathology among the groups. However, all of the patients in IT only group had recurrent metastatic disease, and they were more likely to have received prior definitive therapies.

**Figure 1:**
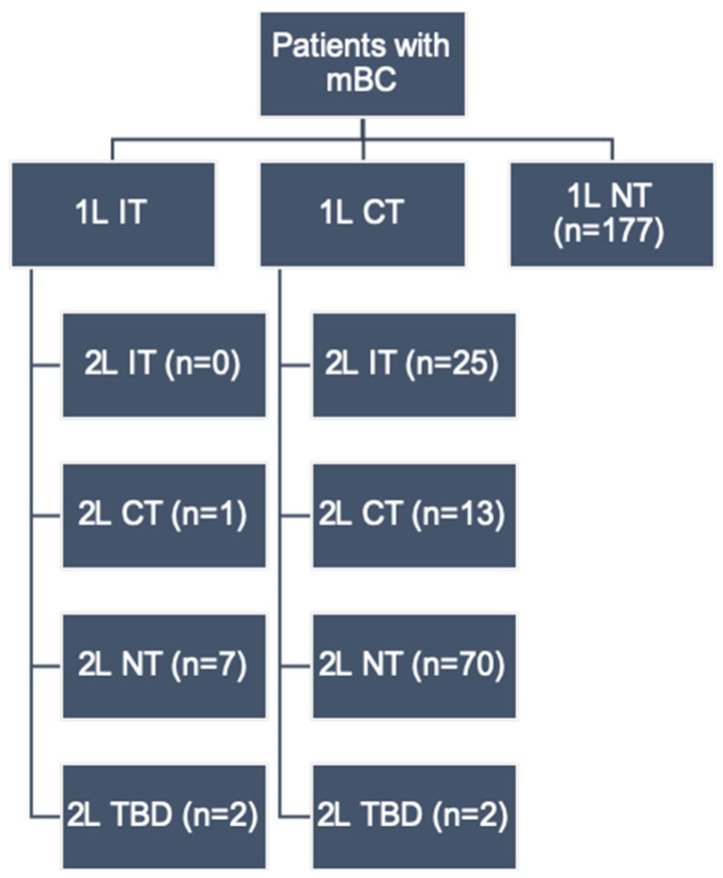
Palliative systemic therapy by line. Abbreviations: mBC = metastatic bladder cancer, 1L = first-line, 2L = second-line, CT = chemotherapy, IT = immunotherapy, NT = no treatment, TBD = to be decided (i.e., patient has not completed 1L therapy or was being considered for 2L therapy at the time of data collection).

Figure 1 summarizes the pattern of treatments in our patient cohort. Of the 297 patients with metastatic disease, 40.4% received 1L PSTs, including chemotherapy (37.0%) and immunotherapy (3.4%), whereas 59.6% received no systemic treatment. Of the 116 patients who received 2L therapy, 12.1% received chemotherapy, 21.6% immunotherapy and 66.4% did not receive any further systemic treatment. Table A1 summarizes the palliative therapies used as first- and second-line agents in this study.

**Figure 2:**
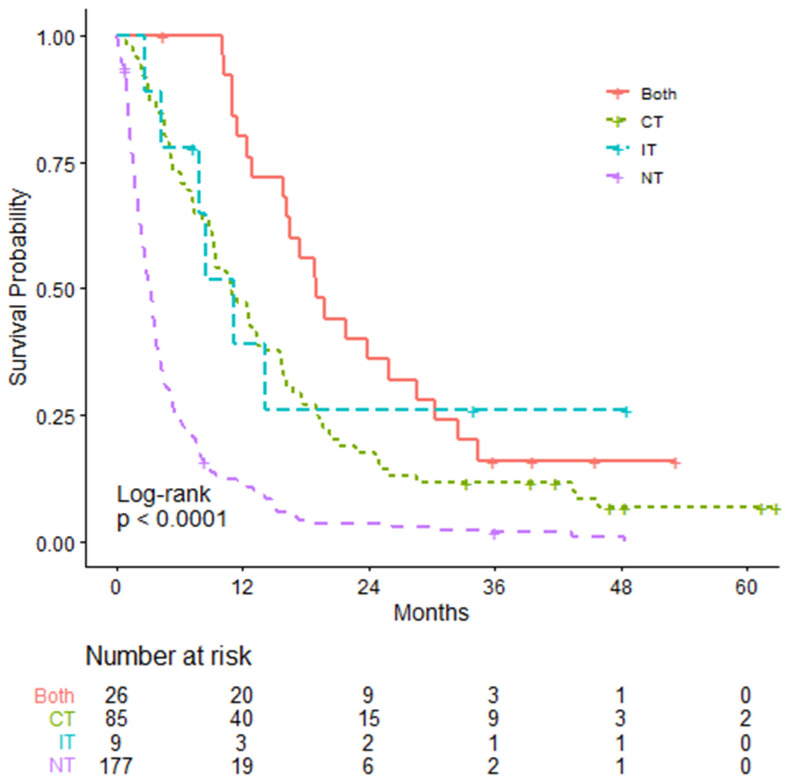
Overall survival in patients receiving palliative therapies, calculated from date of metastasis. Chemotherapy (CT), immunotherapy (IT), chemotherapy and immunotherapy (both) or no treatment (NT).

Table 2 summarizes survival outcomes based on treatment modalities. Patients who did not receive any palliative systemic therapies had mOS of 3.16 months. Among the patients receiving treatment, those receiving both CT and IT had the longest survival with mOS of 18.99 months (HR: 0.231; *p* < 0.0001). In comparison, the CT only and IT only groups had mOS of 10.82 (HR: 0.362; *p* < 0.0001) and 11.10 months (HR: 0.296; *p* = 0.004), respectively (Figure 2).

**Figure 3:**
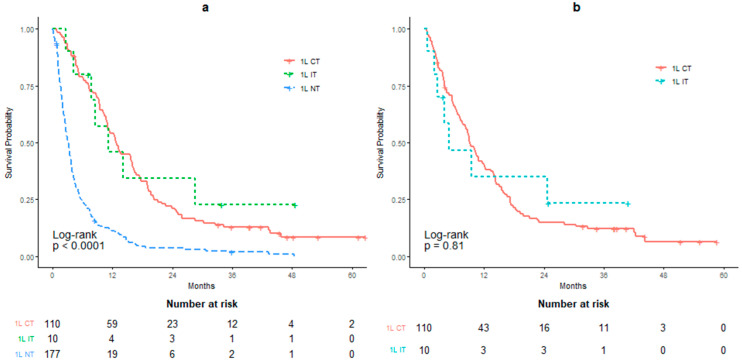
Overall survival in patients receiving first-line palliative therapies. (**a**) OS calculated from date of metastasis. (**b**) OS calculated from initiation of first-line therapy. Chemotherapy (1L CT), immunotherapy (1L IT) or no treatment (1L NT).

**Figure 4:**
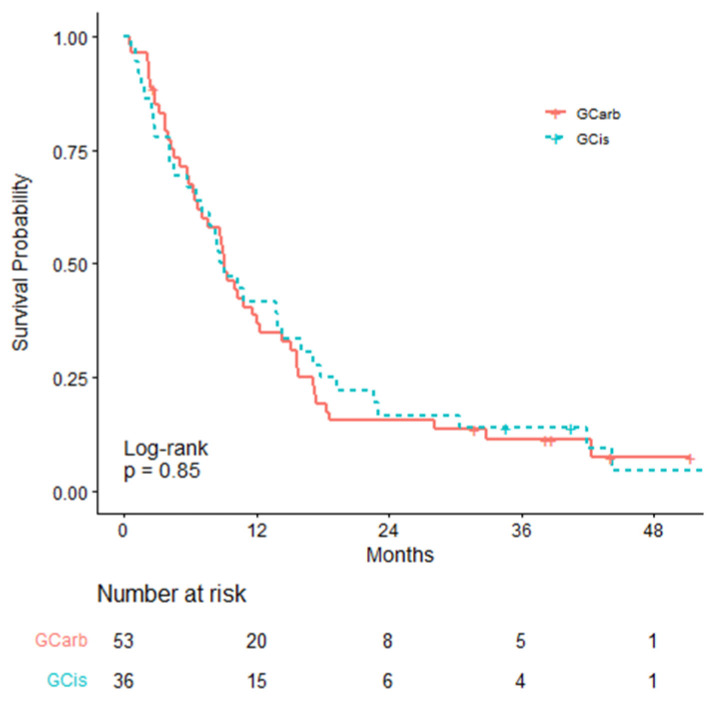
Overall survival in patients receiving first-line palliative chemotherapy, including: gemcitabine and cisplatin (GCis) vs. gemcitabine and carboplatin (GCarb), calculated from initiation of therapy.

The survival for 1L therapy, calculated from time of metastasis, is illustrated in Figure 3a. The mOS for the NT group was significantly shorter compared to either the IT or CT groups (*p* < 0.01; Table A2). When calculated from the treatment start date, the mOS was 5.03 months for the 1L IT group and 9.13 months for the 1L CT group (HR: 0.911; *p* = 0.81; Table A3 and Figure 3b). For the 89 patients receiving 1L CT, 40.4% received GCis and 59.6% received GCarb (Table A4). There was no significant difference (Figure 4) in survival amongst the two groups (GCis mOS: 8.88 months, GCarb mOS: 9.13 months, HR: 1.044, *p* = 0.85) when calculated from the treatment start date.

**Figure 5:**
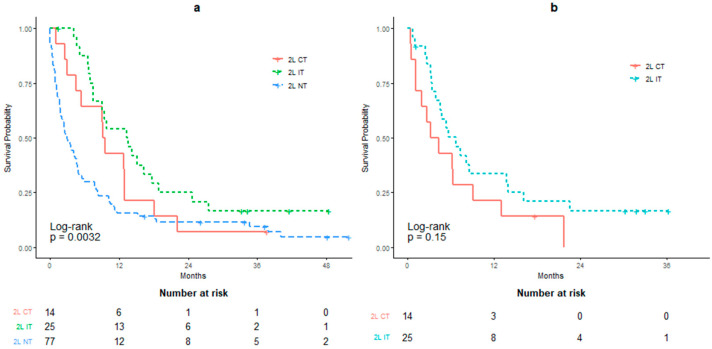
Overall survival (OS) in patients receiving second-line palliative therapies. (**a**) OS calculated from termination of first-line therapy. (**b**) OS calculated from initiation of second-line therapy. Therapies include chemotherapy (2L CT), immunotherapy (2L IT) or no treatment (2L NT).

Figure 5a and Table A5 compare the OS between 2L therapies versus NT. Calculated from the date that 1L agent was terminated, the mOS for the 2L NT group was 2.92 months, whereas the mOS for the 2L IT and CT groups were 13.43 and 9.34 months, respectively. The difference in mOS between 2L IT and 2L NT was significant (HR: 0.454, *p* = 0.0047), but 2L CT to 2L NT comparison did not reach statistical significance (HR: 0.639, *p* = 0.1734). When calculated from the date that 2L therapy was initiated, the mOS for the 2L IT and CT groups were 6.72 months and 3.78 months, respectively (HR: 0.595; *p* = 0.15; Figure 5b and Table A6).

## 4. Discussion

This study outlines observational data on treatment patterns and survival outcomes in patients with metastatic bladder cancer in a multicentre provincial population study. In this study, the majority of patients undergoing first-line PSTs received chemotherapy (91.7%) and a smaller portion received immunotherapy (8.3%). Real-world data on the use of chemotherapy versus immunotherapy in 1L settings are limited. However, 1 study reporting on a cohort of cisplatin-ineligible patients had similar results to our study as patients predominantly received carboplatin-based chemotherapy (76%) over immunotherapy (24%) [10]. In our study, the most commonly used chemotherapy regimens in the 1L setting were GCarb and GCis and the preferred 1L immunotherapy regimens were atezolizumab and pembrolizumab. In the 2L setting, however, the proportion of patients receiving immunotherapy (mainly atezolizumab and pembrolizumab) and chemotherapy (primarily carboplatin-based combination therapies) was 64.1% and 35.9%, respectively. Of note, in 2L settings, atezolizumab was more commonly utilized than pembrolizumab. This is because at the time of our data collection (2014–2016), there was limited evidence supporting the use of either of these agents in mBC as well as more accessibility to atezolizumab through patient assistance programs offered in British Columbia. Importantly, more than half of the patients with mBC did not receive any PST and almost two-thirds of individuals did not undergo 2L treatment. In comparison, 2 recent retrospective studies looking at uptake of chemotherapy in the US, reported 36% of patients did not receive 1L, and 45–66% did not receive 2L systemic therapies [11,12].

Taken from the date of diagnosis of metastasis, in individuals receiving PSTs the mOS was 11.10 and 10.82 months for the IT and CT groups, respectively. Those who received both CT and IT sequentially had a longer mOS of 18.92 months. Conversely, patients not receiving treatment had a mOS of 3.16 months. This difference in survival outcomes was expected as other studies in the literature report an average survival of 9–11 months versus 3–6 months with and without treatment, respectively [7,10,11]. This provides support for placing patients on at least one line of PST in the metastatic setting. Furthermore, a real world mOS of over a year and a half is unprecedented and indicates the efficacy of multiple lines of therapy. To our knowledge, this is the first study comparing survival outcomes retrospectively in a patient cohort receiving immunotherapy to patients not receiving any PSTs.

Taken from the date of 1L therapy initiation, patients receiving 1L CT had an mOS of 9.13 months, which was slightly shorter than those reported by other retrospective studies (11–12.8 months) [11,13]. In contrast, the mOS for 1L IT was approximately half of 1L CT at 5.03 months. The difference in survival was not significant possibly due to the small sample size of the IT group. The limited number of patients in the IT group reflects the recent approval of immune checkpoint inhibitors in 2017 by the FDA for use in 1L setting in cisplatin-ineligible patients. The mOS for 1L IT, taken from the date of 1L therapy initiation, was shorter than those reported in other retrospective (9 months) and prospective clinical trials (15.9 months) [10,14]. This may reflect patient selection of those who received IT as 1L, including those who have poorer functional status due to multiple comorbidities.

Among 1L platinum-based chemotherapies, GCis is currently the recommended first-line regimen according to guidelines. GCarb is reserved for individuals who are GCis ineligible [6]. There is only one published randomized control trial (RCT) which investigated GCis versus GCarb; however, due to a small sample size, this study did not have sufficient power to reliably compare the efficacy of these treatments head-to-head [15]. In our patient cohort, patients receiving GCis had an mOS of 8.88 months, which is shorter than those reported in both retrospective (13.3 months) and RCTs (12.7–14.0 months) [11,15,16,17]. Conversely, individuals receiving GCarb had an mOS of 9.13 months, which is consistent with the literature (9.3–10.6 months) [11,15,18]. There was no significant difference in the mOS between these two groups despite patients on GCarb likely having a number of comorbidities and/or poor performance status rendering them unfit for GCis therapy. This finding is consistent with the literature in both retrospective and RCT settings [11,15]. Given the comparable efficacies of these two regimens, GCarb could be considered a suitable alternative to GCis for 1L CT.

Calculated from the date that 1L agent was terminated, patients in the 2L NT group had an mOS of only 2.92 months, which is similar to the results of another retrospective study (2.8 months) and slightly less than that reported by a clinical trial (4.3 months) [19,20]. Although the mOS of 2L CT (9.34 months) was found to be more than triple that of the 2L NT group, the difference was not statistically significant, potentially due to the small sample size of the CT group. Conversely, the mOS of 2L IT was significantly longer at 13.43 months, demonstrating the importance of immunotherapy access beyond the 1L setting. This benefit is also expected to be translated into the 1L setting in the future once the maintenance atezolizumab treatment following the 1L CT is approved as an option. In our cohort, none of the patients received maintenance IT.

Calculated from the date of 2L therapy initiation, patients receiving CT had an mOS of 3.78 months, which is shorter than those reported by other retrospective studies (6.83–9.4 months) and clinical trials (6–7 months) [13,20,21,22]. Similarly, patients on 2L IT had an mOS of 6.72 months, which is shorter than survival outcomes reported by recent RCTs (8.74 months for nivolumab, 10.1 months for pembrolizumab and 11.1 months for atezolizumab) [23,24,25]. The difference between 2L IT and CT in our study was not found to be significant (*p* = 0.15). The survival benefits of 2L CT remain controversial compared to 2L IT based on recent reports from RCTs. The phase III KEYNOTE-045 study demonstrated a significant benefit in mOS for 2L pembrolizumab versus standard-of-care CT. Conversely, the phase III IMvigor211 study did not report any significant improvements in mOS for 2L atezolizumab versus CT [24,25]. To our knowledge, a retrospective study has never been conducted to directly compare 2L IT to either 2L CT or supportive care for patients. Our results can therefore help inform decisions regarding the use of immunotherapy as a 2L agent in a real-world setting.

There are several limitations to this study. Firstly, the location(s) of the initial metastatic disease was not collected in this study, and therefore its impact on our survival outcomes is unknown. The discussed immune checkpoint inhibitors were only recently approved by Health Canada in 2016 or later, thus limiting our sample size as many patients did not have access to these treatments during the time that the study was conducted. Furthermore, this study was solely based on a single provincial database in Canada. Therefore, there may be a limited applicability of our findings across different populations. In addition, this study was on patients with bladder cancer and did not include less common sites for urothelial carcinoma such as the ureter or renal pelvis. Lastly, this study did not include novel treatment approaches such as combination immunotherapy or targeted therapies with agents such as erdafitinib and enfortumab vedotin. Future studies with larger patient cohorts and longer follow-up times are warranted to rigorously evaluate and shed more light on the efficacy and safety of novel palliative systemic therapies in the real-world setting.

## 5. Conclusions

In summary, we have provided real-world information on treatment patterns and survival outcomes of palliative systemic therapies in treating metastatic bladder cancer. Despite the fact that chemotherapy remains the most widely used treatment modality in first-line setting, our study found immunotherapy to be a comparable alternative. This supports the use of immune checkpoint inhibitors as 1L agents in select patients. Within 1L chemotherapy agents, guidelines currently recommend GCis over GCarb. Our study, however, showed that GCarb may be as efficacious. Additionally, this study provided further evidence for the effectiveness of immunotherapy in second-line settings.

## Figures and Tables

**Table 1:** Patient, disease, and treatment characteristics of the overall cohort.

Variable		Overall (*N* = 297)
Gender (Male), number of patients		228 (77%)
Age at metastasis in years, median (range)		73 (36–98)
Grade, number of pts (%)	High	272 (92%)
	Intermediate	7 (3%)
	Low	5 (2%)
	Unknown	13 (4%)
Histology, number of pts (%)	Urothelial	231 (78%)
	Squamous	5 (2%)
	Neuroendocrine	14 (4%)
	Glandular	2 (<1%)
	Mixed	44 (15%)
	Undifferentiated/other	1 (<1%)
Lymphovascular invasion, number of pts (%)		108 (36%)
Stage at initial diagnosis, number of pts (%)	II	75 (25%)
	III	91 (31%)
	IV	130 (44%)
Radical cystectomy, number of pts (%)		137 (46%)
Radical radiotherapy, number of pts (%)		27 (9%)
Prior curative chemotherapy use, number of pts (%)		73 (25%)

**Table 2:** Survival outcomes in patients receiving palliative therapies, calculated from date of metastasis.

Overall Survival	IT Only *N* = 9	CT Only *N* = 85	Both CT & IT *N* = 26	NT (Reference) *N* = 177
mOS (95% CI; months)	11.10 (7.79–NA)	10.82 (9.26–13.59)	18.99 (16.23–30.26)	3.16 (2.69–3.66)
Log-rank test *p*-value	0.004	<0.0001	<0.0001	-
HR (95% CI)	0.296 (0.13–0.67)	0.362 (0.27–0.48)	0.231 (0.15–0.37)	-
Non-proportionality *p*-value	0.29	0.0002	0.0001	-
RMST (95% CI; months)	18.61 (6.31–30.91)	15.07 (12.27–17.87)	23.68 (18.73–28.63)	5.46 (4.33–6.59)
RMST difference (95% CI; months)	13.15 (0.80–25.50)	9.60 (6.58–12.62)	18.22 (13.14–23.29)	-
RMST difference *p*-value	0.037	<0.0001	<0.0001	-

IT: immunotherapy; CT: chemotherapy; NT: no treatment. mOS: median overall survival. HR: hazard ratio. CI: confidence interval. RMST: restricted mean survival time; restricted at 48 months (4 years).

## Data Availability

Data for this study was collected from the BC Cancer Agency database, which is not publicly available.

## Appendix A

**Table A1:** Palliative systemic therapy by line.

Agents	1L (*N* = 297), *n* (%)	2L (*N* = 116), *n* (%)
**Chemotherapy**	110 (37.0)	14 (12.1)
Gemcitabine + Carboplatin	53 (17.8)	2 (5.2)
Gemcitabine + Cisplatin	36 (12.1)	0 (0.0)
Taxanes alone	2 (0.7)	7 (6.0)
Other ^a^	19 (6.4)	5 (4.3)
**Immunotherapy**	10 (3.4)	25 (21.6)
Atezolizumab	4 (1.3)	16 (13.8)
Pembrolizumab	4 (1.3)	9 (7.8)
Nivolumab	2 (0.7)	0 (0.0)
**No chemotherapy or immunotherapy**	177 (59.6)	77 (66.4)

Abbreviations: 1L = first-line, 2L = second-line. ^a^ Other: in 1L, included cisplatin/etoposide, carboplatin alone, cisplatin alone, gemcitabine alone, carboplatin/etoposide, carboplatin/vinblastine and cyclophosphamide/doxorubicin/vincristine (CAV); in 2L, included pemetrexed, topotecan, carboplatin/irinotecan and carboplatin/5-flurouracil.

**Table A2:** Median overall survival from time of metastasis for first-line palliative systemic treatments versus no treatment.

Overall Survival	1L IT *N* = 10	1L CT *N* = 110	NT (Reference) *N* = 177
mOS (95% CI; months)	11.10 (7.79–NA)	12.76 (10.95–15.59)	3.16 (2.69–3.66)
Log-rank test *p*-value	0.0011	<0.0001	-
HR (95% CI)	0.291 (0.136–0.624)	0.310 (0.239–0.402)	-
Non-proportionality *p*-value	0.2	<0.0001	-
RMST (95% CI; months)	19.686 (8.619–30.753)	16.909 (14.373–19.445)	5.462 (4.333–6.591)
RMST difference (95% CI; months)	14.224 (3.099–25.348)	11.446 (8.670–14.222)	-
RMST difference *p*-value	0.012	<0.0001	-

1L CT: first-line chemotherapy; 1L IT: first-line immunotherapy; NT: no treatment mOS: median overall survival; HR: hazard ratio CI: confidence interval; RMST: restricted mean survival time; restricted at 48 months (4 years).

**Table A3:** Survival outcomes from start date of therapy for first-line palliative immunotherapy and chemotherapy.

Overall Survival	1L IT *N* = 10	1L CT (Reference) *N* = 110
mOS (95% CI; months)	5.03 (2.69–NA)	9.13 (7.72–12.10)
HR (95% CI)	0.911 (0.422–1.967)	-
Log-rank test *p*-value	0.81	-

1L CT: first-line chemotherapy; 1L IT: first-line immunotherapy; NT: no treatment. mOS: median overall survival. HR: hazard ratio. CI: confidence interval.

**Table A4:** Survival outcomes in patients receiving first line palliative chemotherapy including: gemcitabine and cisplatin (GCis) vs. gemcitabine and carboplatin (GCarb).

Overall Survival	GCarb *N* = 53	GCis (Reference) *N* = 36
mOS (95% CI; months)	9.13 (6.69–14.30)	8.88 (6.53–16.10)
HR (95% CI)	1.044 (0.668–1.633)	-
Log-rank test *p*-value	0.85	-

GCarb: Gemcitabine/carboplatin; GCis: Gemcitabine/cisplatin. mOS: median overall survival. HR: hazard ratio. CI: confidence interval.

**Table A5:** Median overall survival from termination of first-line therapy for second-line palliative systemic treatments versus no treatment.

Overall Survival	2L IT *N* = 25	2L CT *N* = 14	2L NT (Reference) *N* = 77
mOS (95% CI; months)	13.43 (9.10–18.82)	9.34 (5.46–22.10)	2.92 (1.85–4.82)
Log-rank test *p*-value	0.0047	0.1734	-
HR (95% CI)	0.454 (0.275–0.749)	0.639 (0.352–1.160)	-
Non-proportionality *p*-value	0.0002	0.0075	-
RMST (95% CI; months)	15.95 (11.67–20.24)	11.36 (6.66–17.87)	7.65 (5.20–10.09)
RMST difference (95% CI; months)	8.31 (3.38–13.24)	3.71 (−1.59–9.00)	-
RMST difference *p*-value	0.001	0.170	-

2L CT: second-line chemotherapy; 2L IT: second-line immunotherapy; NT: no treatment. mOS: median overall survival. HR: hazard ratio. CI: confidence interval. RMST: restricted mean survival time; restricted at 36 months (3 years).

**Table A6:** Survival outcomes from start date of therapy for second-line palliative immunotherapy and chemotherapy.

Overall Survival	2L IT *N* = 25	2L CT (Reference) *N* = 14
**mOS** **(95% CI; months)**	6.72 (4.59–16.10)	3.78 (1.99–NA)
**HR** **(95% CI)**	0.595 (0.293–1.209)	-
**Log-rank test** ***p*-value**	0.15	-

2L CT: second-line chemotherapy; 2L IT: second-line immunotherapy. mOS: median overall survival. HR: hazard ratio. CI: confidence interval.
